# AI-Enabled Hardware-in-the-Loop Validation for Automotive Cybersecurity: A Review of Cyber Threats, Testbeds, and Intelligent Detection

**DOI:** 10.3390/s26185840

**Published:** 2026-09-15

**Authors:** Farshideh Kordi, Paul Fortier, Amine Miled

**Affiliations:** Department of Electrical and Computer Engineering, Université Laval, Québec, QC G1V 0A6, Canada; paul.fortier@gel.ulaval.ca

**Keywords:** Hardware-in-the-Loop (HIL) simulation, electronic control unit (ECU), cyberattacks, artificial intelligence (AI), cybersecurity

## Abstract

Cybersecurity has become one of the most critical challenges in the intelligent and connected vehicle ecosystem of today. As modern vehicles become increasingly connected and intelligent, the frequency and sophistication of cyberattacks targeting automotive systems continue to grow at an alarming rate. Ensuring robust detection and prevention mechanisms has therefore become essential to safeguard driver safety and vehicle integrity. Rapid and accurate identification of cyberthreats is critical, as such attacks can disrupt vital Electronic Control Units (ECUs) and compromise functions such as braking, steering, or communication networks. This review provides a comprehensive analysis of the major categories of cyberattacks targeting modern vehicles, including physical, remote, in-network, firmware- and software-based, cloud- and connectivity-related, and sensor-level perception attacks. Contemporary vehicle architectures, connected-vehicle technologies, software-update mechanisms, and current automotive cybersecurity standards and regulations are also considered. Although traditional cybersecurity testing approaches offer valuable insight into software vulnerabilities, they fail to capture the full cyber–physical interactions that govern vehicle behavior under malicious conditions. In this review, we highlight the essential role of Hardware-in-the-Loop (HIL) and Vehicle-in-the-Loop (VIL) platforms as realistic and safe environments for evaluating the impact of cyberattacks on automotive control systems and for generating synchronized cyber–physical data under controlled attack scenarios. We further examine how artificial intelligence (AI) techniques contribute to detecting, mitigating, and countering these cyberthreats, including supervised and unsupervised intrusion detection, deep-learning-based temporal modeling, cyber–physical anomaly detection, and the emerging challenge of adversarial attacks against AI-based detectors. By synthesizing insights from automotive cybersecurity, HIL-/VIL-based validation, automotive cybersecurity datasets, and AI-driven intrusion detection, this paper establishes a foundation for developing and evaluating more resilient and secure connected and software-defined vehicle architectures.

## 1. Introduction

During the past decade, there has been rapid progress in the electrification and automation of modern vehicles. Modern vehicles comprise networks of numerous electronic control units (ECUs) that provide ubiquitous connectivity and control a wide spectrum of electronic functions [1]. As core components of automotive systems, ECUs manage critical functions such as braking, steering, and engine control, thereby ensuring vehicle safety and performance [2]. As vehicles evolve into highly networked and intelligent systems, ECUs have become more complex, tightly interconnected, and increasingly reliant on real-time data exchange across mechanical, electrical, and cyber domains [3]. In parallel, modern vehicles depend on external interconnected systems such as Vehicle-to-Vehicle (V2V), Vehicle-to-Infrastructure (V2I), and Vehicle-to-Everything (V2X) [4]. These communication technologies enable vehicles to exchange information, improve situational awareness, and support improved driving decisions. Furthermore, modern vehicles integrate satellite communication (for satellite radio and navigation), cellular connectivity, WiFi, and Bluetooth, which are typically managed by an in-vehicle information system or head unit [5], providing occupants with greater comfort and seamless access to vehicle functions. However, this growing interconnectivity also exposes them to a broad spectrum of cybersecurity threats, making security a critical consideration for their deployment and adoption [6]. High-profile incidents such as remote hijacking of vehicle functions, sensor spoofing, and unauthorized intrusion into critical in-vehicle networks have further highlighted the severe risks posed by security breaches in modern connected vehicles [7,8].

The primary goal of cybersecurity testing is to identify these security vulnerabilities and ultimately ensure that the functionality of the system remains secure [9]. Identifying cybersecurity weaknesses effectively requires comprehensive testing across a wide range of representative attack and operating scenarios [1,10]. Cyberattacks targeting automotive systems can lead to severe consequences such as unintended acceleration, steering manipulation, or brake system compromise [11]. However, because full-scale testing on moving vehicles is expensive, risky, and logistically challenging, most vehicle cybersecurity research has relied on simulations, isolated subsystems, or narrowly scoped case studies rather than comprehensive real-vehicle experiments that include realistic human interactions.

In this context, Hardware-in-the-Loop (HIL) simulators are powerful tools that enable the detection of vulnerabilities while maintaining safety and controllability. They provide a robust means for testing and validating both the functional behavior and security aspects of ECUs by replicating real-world operating conditions and disturbances encountered by vehicle subsystems such as braking, suspension, and steering. For example, in [12], a new testing framework was developed combining HIL simulation with real-time fault injection (FI) to validate the performance and robustness of the system under critical abnormal and fault-induced conditions during the development process. Similarly, in [13], a Cyber-HIL platform was proposed as a comprehensive and effective approach to test and validate the security of ship control systems in the presence of cyberthreats, demonstrating the applicability of HIL-based methods to cybersecurity evaluation in safety-critical domains.

Despite their strengths, conventional cybersecurity testing methods—such as penetration testing and static code analysis—are largely manual, rule-based, and signature-driven, and are often inadequate for evaluating ECU resilience against sophisticated, adaptive attack strategies [14]. The emergence of artificial intelligence (AI) and deep learning offers new opportunities to overcome these limitations and enhance the traditional HIL simulation and ECU testing methodologies [15]. As an early example of data-driven system identification, Ogunmolu et al. [16] investigated in an arXiv preprint the use of deep neural networks for modeling nonlinear dynamical systems from sequential input–output data. Consequently, there is a growing need for intelligent, AI-driven cybersecurity testing platforms capable of simulating realistic attack scenarios, detecting anomalies in real time, and quantifying the impact of cyberthreats on vehicle safety and performance. In this context, AI provides powerful mechanisms for anomaly detection, real-time threat analysis, and automated response, enabling more adaptive and scalable automotive security architectures [17].

Given this rapidly evolving and increasingly sophisticated attack landscape, there is an urgent need for new strategies to strengthen vehicle cybersecurity. Machine learning, artificial intelligence, and systematic cybersecurity engineering form key building blocks of such advanced defense approaches. Consequently, the main motivation of this article is threefold: (i) to provide a comprehensive overview of cyberattacks targeting modern vehicles; (ii) to examine the critical role of HIL simulation in detecting and evaluating such threats; and (iii) to review AI-based techniques that are integrated into HIL testbeds for detecting, mitigating, and evaluating automotive cybersecurity attacks.

However, there is a noticeable lack of comprehensive surveys that jointly consider automotive cyberattacks, HIL-based cyber–physical testbeds, and AI-driven security mechanisms at the ECU and embedded-system level. Existing reviews typically address these aspects in isolation—focusing on attack taxonomies, vehicle network security, or AI-based intrusion detection—without systematically connecting them to concrete HIL implementations and hardware-centric validation workflows. This gap limits the community’s ability to design, implement and benchmark realistic AI-enabled cybersecurity evaluation platforms for modern vehicles. We seek to address this gap by presenting a structured review of recent literature on the use of AI for automotive cybersecurity in conjunction with HIL-based and related embedded test environments, thereby providing a timely reference for both researchers and practitioners working toward secure and trustworthy automotive systems.

Given the increasing complexity of automotive cyber–physical systems, evaluating cybersecurity mechanisms requires more than independently studying vehicle attacks, test platforms, and intrusion-detection algorithms. A meaningful security assessment must connect the attack surface of the target vehicle architecture to the physical and network variables affected by an attack, reproduce these effects under realistic operating conditions, and evaluate whether detection mechanisms can identify the resulting cyber–physical deviations, and whether appropriate mitigation or response mechanisms can contain their effects within real-time constraints. HIL and Vehicle-in-the-Loop (VIL) platforms provide an important foundation for such evaluation because they combine real automotive hardware and communication interfaces with controllable virtual vehicle and environment models.

Accordingly, the scope of this review is specifically focused on the intersection of automotive cybersecurity, HIL-/VIL-based cyber–physical validation, and AI-enabled intrusion and anomaly detection. Rather than treating these areas as independent topics, the review examines how contemporary automotive attack surfaces can be represented within HIL/VIL environments, what network, sensor, actuator, and control-system signals can be collected during attack experiments, and how these data can subsequently be used to train, validate, and benchmark AI-based detection mechanisms.

The main contributions of this review are therefore fourfold. First, we examine automotive cyber threats in the context of contemporary vehicle architectures and communication technologies and identify the cyber–physical consequences that should be reproduced during security testing. Second, we analyze HIL, VIL, and related cyber–physical test environments from a cybersecurity perspective, emphasizing their capability to reproduce attacks safely, repeatedly, and under realistic timing and closed-loop operating conditions. Third, we review AI-based intrusion and anomaly-detection approaches with particular emphasis on methods applicable to automotive network traffic, ECU behavior, sensor signals, and HIL-/VIL-generated data. Finally, we synthesize these areas through an attack-to-detection perspective that relates vehicle attack surfaces and threat types to their HIL/VIL representation, observable signals, AI-based detection strategies, and relevant evaluation criteria.

This integrated perspective distinguishes the present review from surveys that address automotive attack taxonomies, vehicle-network intrusion detection, or HIL-based validation independently. The objective is not merely to catalog attacks and AI algorithms, but to provide a structured framework for designing and evaluating realistic AI-enabled automotive cybersecurity test environments capable of connecting cyberattacks to measurable cyber–physical effects and detection outcomes.

### Review Methodology and Scope

This review was conducted to identify and synthesize literature at the intersection of automotive cybersecurity, HIL and VIL validation, and AI-based intrusion and anomaly detection. The literature search considered studies published between 2010 and August 2026, with particular emphasis on research published after 2021 to capture recent developments in automotive E/E architectures, cybersecurity standards, connected-vehicle technologies, HIL/VIL platforms, and AI-based detection methods. Earlier seminal studies were also retained when necessary to provide historical context for established automotive attack mechanisms and communication technologies.

Relevant literature was identified through Google Scholar and targeted searches of IEEE Xplore, ScienceDirect, SpringerLink, and the ACM Digital Library. Search terms covered three main themes: automotive cybersecurity, cyber–physical validation, and AI-based detection. Representative combinations included “automotive cybersecurity”, “CAN intrusion detection”, “hardware-in-the-loop cybersecurity”, “vehicle-in-the-loop cybersecurity”, “automotive digital twin”, and “machine learning automotive cybersecurity”. Additional studies were identified through backward and forward citation screening of relevant publications.

Studies were considered for inclusion when they addressed at least one of the following areas: (i) cybersecurity threats or attack surfaces relevant to modern vehicle architectures; (ii) HIL, VIL, simulation, or digital-twin platforms used for automotive cybersecurity testing or cyber–physical validation; or (iii) AI-based intrusion, anomaly, or attack-detection methods applicable to in-vehicle networks, vehicle sensors, ECUs, connected services, or HIL/VIL-generated data. Priority was given to peer-reviewed journal and conference publications, standards, regulatory documents, and primary technical reports.

Studies were excluded when they were outside the automotive or closely related cyber–physical context, did not provide sufficient technical detail, duplicated previously identified work, or addressed AI, HIL, or cybersecurity without a meaningful connection to the scope of this review. Non-peer-reviewed sources were used selectively when they represented primary technical disclosures, official standards, regulatory documents, or documented automotive cybersecurity incidents.

The selected literature was qualitatively synthesized and organized according to vehicle architecture and attack surface, HIL/VIL representation, observable cyber–physical signals, AI-based detection approach, and relevant evaluation criteria. The final review incorporates 121 unique sources, including peer-reviewed journal and conference publications, standards, regulatory documents, and selected primary technical reports. Because the review was originally conducted as a structured narrative review, record-level screening logs were not maintained during the initial literature search. Consequently, retrospective counts of records initially identified, deduplicated, and excluded could not be reconstructed reliably. Rather than introducing artificial precision, the present revision reports the databases and platforms searched, representative search terms, publication period, inclusion and exclusion criteria, and backward and forward citation-screening procedure to improve the transparency and reproducibility of the review.

The remainder of this paper is organized to progressively connect contemporary automotive cyber threats with their experimental representation and AI-based detection. Section 2 reviews the evolution of vehicle architectures, communication technologies, attack surfaces, and representative cyber threats, with particular attention to developments in connected and software-defined vehicles. Section 3 examines cybersecurity-oriented HIL, VIL, and related digital-twin validation environments, focusing on how attacks and their cyber–physical consequences can be reproduced safely and repeatably. Section 4 reviews AI-based intrusion and anomaly-detection approaches applicable to automotive network traffic, ECU behavior, sensor signals, and data generated through HIL/VIL experimentation. Section 5 integrates these areas by mapping vehicle architectures and attack surfaces to representative threats, HIL/VIL implementations, observable signals, AI detection methods, and evaluation criteria.

## 2. Contemporary Automotive Architectures and Cyber Threats

### 2.1. Evolution of Automotive E/E Architectures

Automotive electrical/electronic (E/E) architectures have evolved substantially as vehicles have incorporated increasingly complex software, advanced driver-assistance functions, connectivity, electrification, and automated-driving capabilities. Traditional vehicle architectures relied on large numbers of distributed ECUs, typically interconnected through multiple domain-specific communication buses. Although this approach remains relevant in the installed vehicle fleet, increasing computational, communication, software-management, and wiring complexity has motivated a transition toward more consolidated E/E architectures [18].

Contemporary architecture development broadly includes both domain-oriented and zone-oriented approaches. In domain-oriented architectures, functions associated with areas such as powertrain, chassis, body, infotainment, or advanced driver-assistance systems are consolidated into domain control units with greater computational capability. Zone-oriented architectures instead organize vehicle electronics according to physical location, allowing nearby sensors and actuators to connect to zonal control units while higher-level processing can be performed by central or high-performance computing platforms. Automotive Ethernet is increasingly used as a high-bandwidth backbone connecting these controllers, although CAN, CAN FD, LIN, and other communication technologies may remain at the edges of heterogeneous in-vehicle networks [18].

This architectural evolution also changes the automotive cybersecurity problem. Traditional distributed E/E architectures typically isolate vehicle functionality across numerous ECUs and communication buses, whereas domain-oriented and zone-oriented architectures consolidate functions and introduce highly connected domain controllers, zonal controllers, gateways, Ethernet backbones, and centralized or high-performance computing resources [18]. These architectures therefore create different communication dependencies and attack paths across previously separated vehicle functions. Modern automotive attack-surface analyses accordingly consider not only individual ECUs and local buses, but also Ethernet backbones, domain gateways, heterogeneous subnetworks, external interfaces, and the paths through which an attacker may reach security-relevant assets [19]. Consequently, cybersecurity evaluation of contemporary vehicles should represent cross-domain communication, heterogeneous network traffic, centralized computing functions, and the interactions between software services and physical vehicle behavior [18,19].

The attack surface of a vehicle consists of all points where an adversary can attempt to inject or extract data to compromise its security. Pekaric et al. [20] group vehicle attack surfaces into three main categories: physical access, proximity, and remote access. Physical access attacks target in-vehicle components and networks, such as the CAN bus, FlexRay, automotive Ethernet, EV charging ports, the OBD-II diagnostic connector, infotainment/media units and ECUs. Proximity attacks are based on short-range wireless channels, including Wi-Fi, Bluetooth, and wireless sensors such as tire pressure monitoring systems. Remote attacks, by contrast, can be launched over long distances via cellular or other mobile networks, and may also exploit GPS and radio-based signals.

Alarmingly, the security technology protecting these ECUs has not kept pace, allowing adversaries to remotely manipulate critical functions such as steering, acceleration, and even braking, thereby endangering occupants. This threat is not merely theoretical, as demonstrated by several high-profile incidents. In 2015, Miller and Valasek demonstrated the remote exploitation of an unaltered Jeep Cherokee through its connected infotainment and telematics interfaces, showing that a remote compromise could propagate to safety-relevant vehicle functions [8].

In 2016, Tencent Keen Security Lab demonstrated a remote attack on a Tesla Model S that enabled control of selected vehicle functions [21]. In 2018, Tencent Keen Security Lab reported an experimental security assessment of multiple BMW vehicles in which fourteen vulnerabilities were identified across the infotainment Head Unit, Telematics Control Unit (T-Box), and Central Gateway Module. The researchers demonstrated both local and remote attack vectors and showed that compromise of connected vehicle components could be used to transmit unauthorized diagnostic requests through the gateway toward ECUs on different CAN buses [22]. Some classic cases of cyberattacks in recent years are summarized in Table 1.

### 2.2. Types of Cyber Threats Targeting Automotive Systems

Vulnerabilities within a vehicle can have severe consequences, ranging from information theft to potentially life-threatening situations. Numerous studies have shown that attackers can gain malicious control over a vehicle by exploiting one or more weaknesses in its software stack. For example, Koscher et al. [24] demonstrated that an adversary with access to a car’s internal network can manipulate its behavior, including safety-critical functions such as engaging or disabling the brakes.

Modern vehicles are exposed to a wide spectrum of cyberthreats due to increased connectivity, complex software architectures, and reliance on distributed ECUs. These threats can originate from physical access points, wireless communication interfaces, internal vehicle networks, cloud and backend services, software and update mechanisms, or sensor-level perception systems. This section categorizes and explains the main cyberthreats that target automotive systems.

#### 2.2.1. In-Network Attacks

In-vehicle networks (IVNs) specify the connectivity standards that enable reliable and efficient communication among ECUs within a vehicle, supporting the exchange of both data and control signals. Vital information is continuously shared among the numerous ECUs integrated into modern vehicles, and as their number increases, the complexity of IVNs grows accordingly due to the diverse bandwidth, latency, and reliability requirements of different automotive subsystems. To accommodate this heterogeneity, a variety of IVN communication protocols have been developed, and ongoing research continues to refine their performance, efficiency, and security [25].

**Figure 1:**
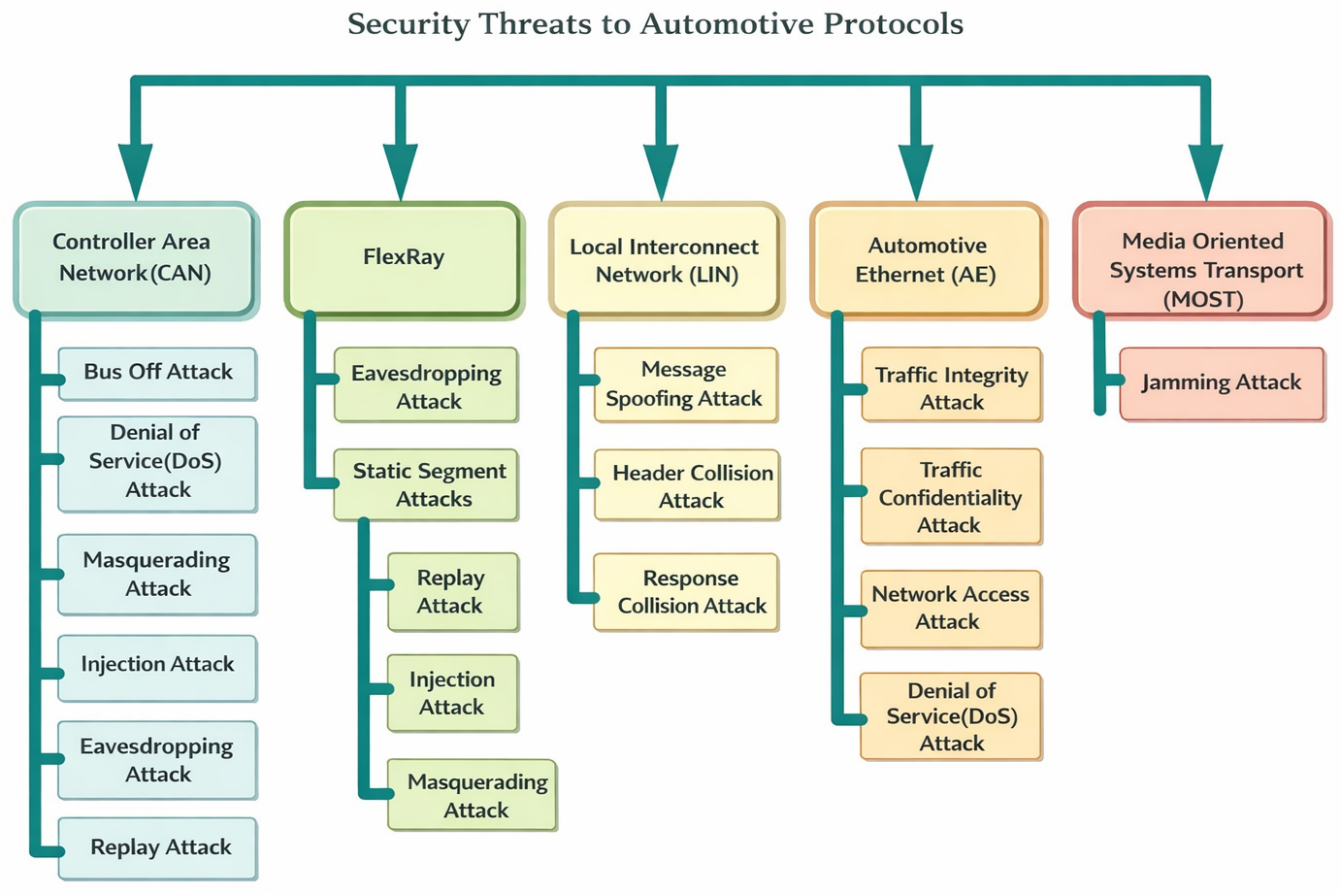
Classification of security threats across major in-vehicle network (IVN) protocols.

Contemporary in-vehicle networks are heterogeneous and may combine Classical CAN, CAN FD, CAN XL, LIN, and Automotive Ethernet according to bandwidth, latency, cost, and functional requirements, while legacy technologies such as FlexRay and MOST remain relevant to earlier and installed vehicle architectures [18,26]. Because each of these protocols has distinct communication characteristics, they exhibit different cybersecurity vulnerabilities [25]. As illustrated in Figure 1, CAN networks are especially prone to bus-off states, denial-of-service (DoS) flooding, spoofing, and message injection attacks, largely due to the absence of built-in authentication and encryption mechanisms.

**Controller Area Network: Classical CAN, CAN FD, and CAN XL**

The Controller Area Network (CAN) family remains a fundamental communication technology in automotive E/E architectures, but its capabilities have evolved substantially beyond the original Classical CAN protocol. Classical CAN provides priority-based, event-triggered communication using non-destructive bitwise arbitration and supports payloads of up to eight bytes per data frame. CAN with Flexible Data Rate (CAN FD) extends this design by permitting larger payloads and a higher transmission rate during the data phase while retaining the arbitration principles and compatibility concepts of Classical CAN. More recently, CAN XL further extends the protocol family to support substantially larger data fields and higher-throughput communication. ISO 11898-1:2024 specifies implementation options supporting Classical CAN, CAN FD, and CAN XL, with CAN XL frames providing data fields of up to 2048 bytes [26].

Despite these protocol extensions, contemporary vehicles generally employ heterogeneous networks rather than replacing Classical CAN uniformly with a single newer protocol. Classical CAN, CAN FD, LIN, Automotive Ethernet, and increasingly CAN XL may coexist according to bandwidth, latency, cost, and functional requirements [18,26]. Communication among these networks is typically mediated by gateways, domain controllers, or zonal controllers, which can translate or route information between different vehicle segments. Consequently, the cybersecurity implications of CAN can no longer be considered solely at the level of an isolated bus: compromise of a CAN-connected ECU or interface may interact with gateway functions and other in-vehicle networks, creating attack paths across multiple communication domains [19].

**Figure 2:**
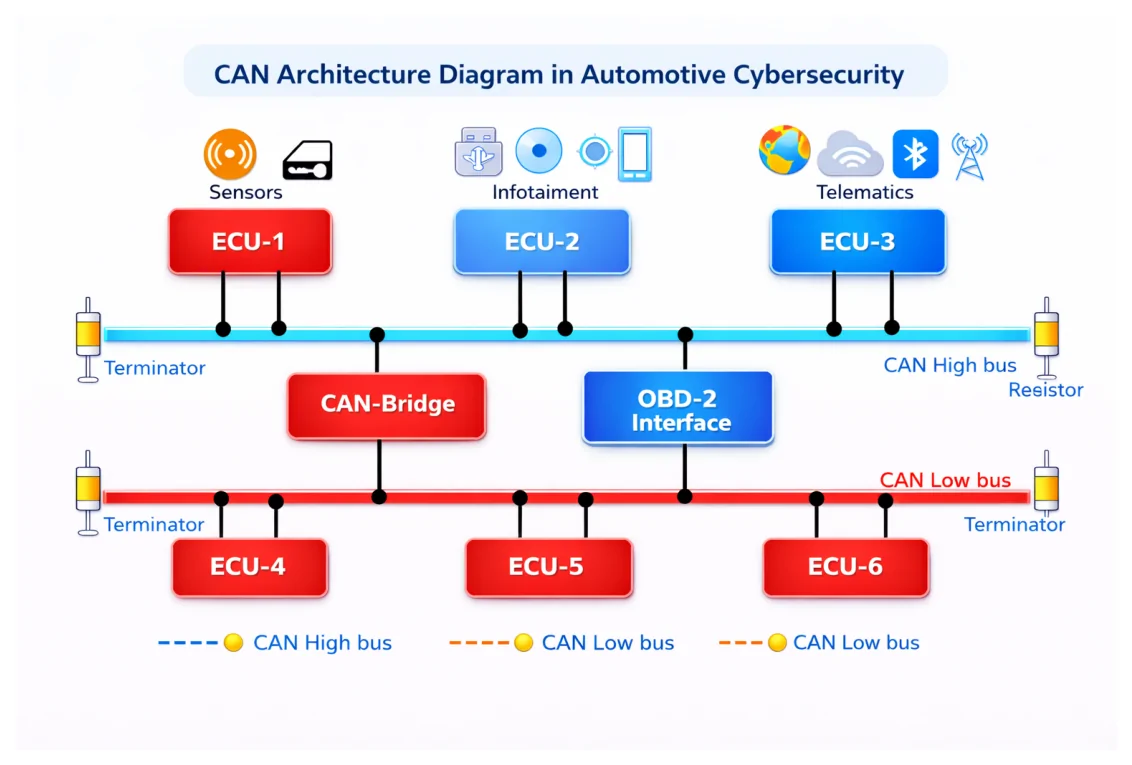
Representative distributed CAN architecture with two CAN segments interconnected through a gateway and an OBD-II diagnostic interface. Such architectures remain relevant in the installed vehicle fleet but are increasingly complemented by domain-, zonal-, and Ethernet-based E/E architectures.

Figure 2 illustrates a representative distributed CAN architecture with two CAN segments interconnected through a gateway and an OBD-II diagnostic interface.

At the protocol level, the CAN family does not inherently provide cryptographic message authentication or confidentiality, so security mechanisms must be implemented at higher layers or through complementary architectural controls. Because CAN frames are typically unencrypted, an adversary with bus access can passively monitor traffic (eavesdropping) and potentially collect sensitive information (e.g., location data or infotainment-related data). In addition, the attacker can actively inject forged CAN frames to influence ECU behavior [27]. Prior work commonly reports six major attack types targeting CAN: bus-off, denial-of-service (DoS), masquerading, injection, eavesdropping, and replay attacks [28]. Among these, DoS attacks are particularly frequent in practice, as high-priority flooding can starve legitimate traffic.

- Masquerading attack: The attackers can gain access to CAN frames due to the lack of encryption and message authentication, making it easier for them to infiltrate the network. The adversary first suppresses a legitimate message and then injects counterfeit frames that mimic its behavior, using the same transmission intervals, message format, and payload value ranges [29].
- Eavesdropping attack: The attackers can eavesdrop on broadcasted vehicular CAN messages, potentially allowing them to infiltrate in-vehicle networks.
- Injection attack: The attackers may attempt to inject false signals into the vehicle’s bus system. Using on-board diagnostics (OBD) ports, they can establish connections with the in-vehicle system, potentially compromising the ECUs.
- Replay attack: The attackers can disrupt the vehicle’s real-time operation by continuously retransmitting legitimate frames. Mitigating replay attacks is a challenging task because network entities often cannot determine whether they are under attack [30].
- Bus-off attack: The attacker may continuously send data bits not only in the identification field but also in other fields.
- DoS attack: The attacker may disrupt the normal processing of in-vehicle communication by continuously sending high-priority CAN packets, which can block valid packets with low-priority and potentially gain control of the vehicle [31].

**Local Interconnect Network (LIN) bus**

LIN is a one-wire network designed to connect sensors and actuators. LIN’s reliability falls short compared to CAN, making it unsuitable for time-critical applications [32]. Moreover, due to the limited data transmission capacity, it is not feasible to implement it in a high-speed communication system [33]. Three attacks usually occur in LIN, namely message spoofing, header collision, and response collision attacks.

**FlexRay**

The FlexRay protocol was originally introduced by BMW in 2007 as a high-speed in-vehicle communication protocol that uses two parallel channels to support both synchronous (time-triggered) and asynchronous (event-triggered) data transmission. Each channel provides data rates of up to 10 Mbps, offering significantly higher bandwidth than legacy IVNs such as CAN and LIN and enabling the concurrent delivery of time-critical control messages and event-driven traffic. This dual-channel architecture improves reliability and fault tolerance, but increased implementation cost and system complexity have limited the widespread deployment of FlexRay in production vehicles [34]. Eavesdropping and static-segment attacks are the two main security threats to FlexRay. To counteract them, advanced authentication mechanisms are used to verify messages within the static segment as a preventive measure [35].

**Media-Oriented Systems Transport (MOST)**

The MOST protocol, a domestic digital bus, supports both synchronous and asynchronous modes for data transmission. In the context of MOST, two common types of attack that can occur are jamming attacks and synchronization disruption attacks. Synchronization disruption attacks involve the hacker attempting to interfere with the MOST network’s synchronization by continuously sending fake timing frames. In jamming attacks, the hacker attempts to disrupt the MOST network by continuously transmitting deceptive messages, specifically targeting low-priority legitimate messages with specified lengths [36]. MOST has been used by renowned car manufacturers, such as BMW, Mercedes-Benz, Porsche, Audi, Volkswagen, Jaguar, Hyundai, Toyota, Land Rover, and many others [33].

**Automotive Ethernet (AE)**

Automotive Ethernet has become an important high-bandwidth communication technology for contemporary in-vehicle networks, particularly as vehicle E/E architectures evolve toward domain-oriented, zonal, and centralized computing platforms. Unlike traditional shared automotive buses, Ethernet uses switched communication and can provide scalable backbone connectivity among domain controllers, zonal controllers, high-performance computing platforms, sensors, infotainment systems, and gateways. Consequently, Ethernet increasingly complements rather than completely replaces legacy protocols such as Classical CAN, CAN FD, and LIN within heterogeneous vehicle networks [18].

The adoption of Ethernet also changes the cybersecurity characteristics of the in-vehicle network. Ethernet-based architectures introduce technologies and protocol layers that are closely related to conventional IP networking, thereby expanding the attack surface beyond message manipulation on a local automotive bus. Potential threats include spoofing, denial-of-service, manipulation of network traffic, attacks on switching or routing functions, and compromise of higher-layer services. Furthermore, because Ethernet backbones interconnect multiple vehicle domains, successful compromise of a highly connected component or gateway may provide attack paths toward otherwise separated vehicle functions [19,37].

For cybersecurity-oriented HIL/VIL evaluation, this transition implies that representative test environments should increasingly reproduce not only CAN traffic and ECU-level behavior but also switched Ethernet communication, gateway interactions, heterogeneous protocol traffic, and, where relevant, IP- and service-oriented communication. Such capabilities are necessary to evaluate attacks that propagate across multiple communication layers and vehicle domains in contemporary E/E architectures [18,37].

**AUTOSAR, Service-Oriented Communication, and Security Gateways**

The evolution toward domain-oriented, zonal, and centralized E/E architectures is accompanied by substantial changes in the automotive software architecture. AUTOSAR distinguishes between the Classic Platform and the Adaptive Platform. The Classic Platform follows a layered software architecture comprising the application layer, Runtime Environment (RTE), and Basic Software (BSW), and is primarily intended for deeply embedded ECUs requiring predictable and resource-constrained execution. In contrast, the Adaptive Platform targets high-performance ECUs and highly automated vehicle functions and provides service- and API-based interfaces together with support for dynamic software updates and reconfiguration [38,39].

Service-oriented communication is particularly relevant to contemporary Automotive Ethernet architectures. AUTOSAR defines Scalable service-Oriented MiddlewarE over IP (SOME/IP) as an automotive communication protocol supporting remote procedure calls, event notifications, and inter-ECU client–server communication. Such service-oriented mechanisms enable flexible interaction among applications distributed across high-performance computing platforms, domain controllers, and other ECUs. However, they also extend the cybersecurity problem from manipulation of individual bus frames toward attacks on network services, service discovery, communication endpoints, and inter-domain data exchange [37,40].

Security gateways and network-segmentation mechanisms therefore play an increasingly important role in contemporary E/E architectures. AUTOSAR defines requirements for Automotive Ethernet firewalls that control communication between ECUs or network zones according to security policies and access-control rules. AUTOSAR also specifies an Intrusion Detection System Manager (IdsM) for collecting, qualifying, and managing security events generated by automotive software components [41,42]. From a cybersecurity-validation perspective, HIL/VIL environments representing modern vehicles should consequently consider not only ECU and network behavior but also gateway filtering, cross-domain communication, service-oriented traffic, and security-event monitoring. This enables experimental platforms to evaluate whether an attack is blocked, propagated across network zones, or detected by security mechanisms before producing an unacceptable cyber–physical effect.

#### 2.2.2. External Network Attacks

**Figure 3:**
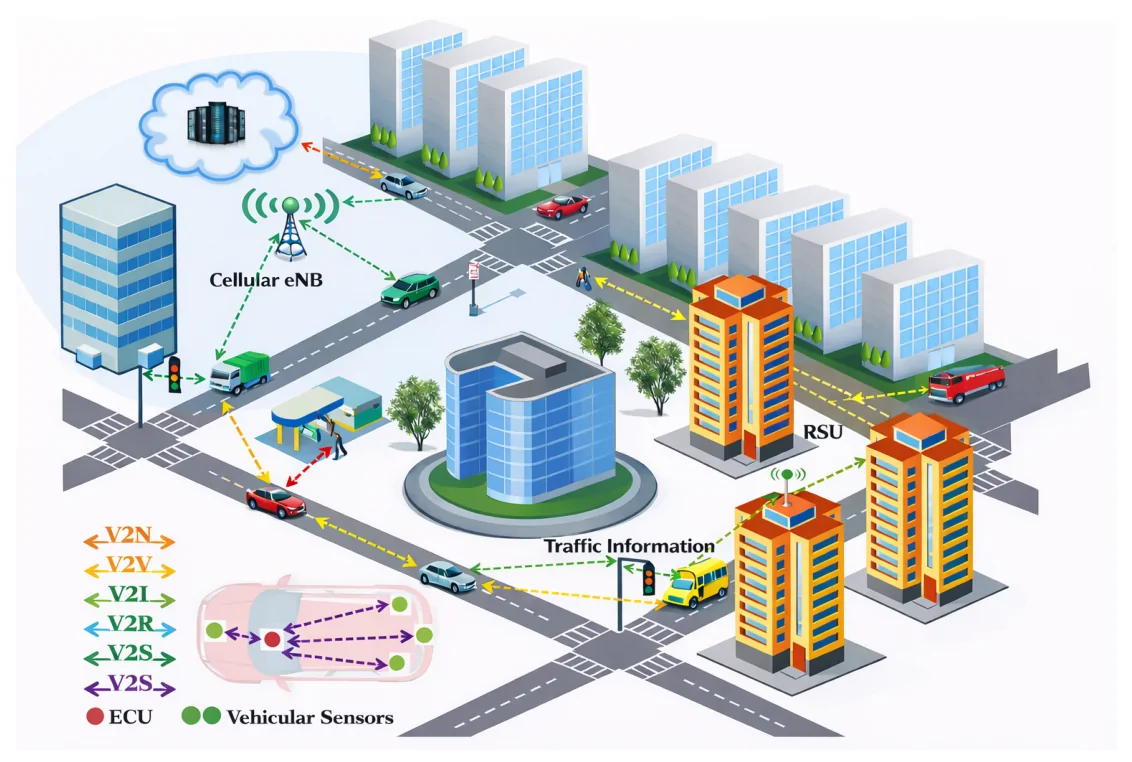
Vehicle-to-anything communication.

Advanced wireless technologies enable vehicles to exchange real-time information with their surroundings, with the potential to reduce accidents, alleviate traffic congestion, and reduce greenhouse gas emissions. As illustrated in Figure 3, wireless access technologies support various communication modes, including vehicle-to-vehicle (V2V), vehicle-to-infrastructure (V2I), vehicle-to-network (V2N), vehicle-to-device (V2D), vehicle-to-pedestrian (V2P), and more generally vehicle-to-everything (V2X). However, as vehicle connectivity to external networks increases, the exposure to cyberthreats also grows, making external network attacks a significant concern [43].

V2X communication enables continuous data exchange between vehicles and surrounding infrastructure elements (e.g., traffic lights, road signs, and roadside units), thus supporting coordinated traffic management and safety applications. However, these wireless links are vulnerable to spoofing attacks in which adversaries inject falsified messages into the V2X network. In such a scenario, a vehicle may receive deceptive warnings about obstacles or phantom vehicles, leading to inappropriate emergency braking or abrupt evasive maneuvers [44].

Each vehicle is typically associated with a unique identifier that is used to distinguish both the vehicle itself and the messages it transmits. Communication between a host vehicle and nearby vehicles during maneuvers such as overtaking, lane changes, and intersection crossing is typically carried over V2V links. This inter-vehicle connectivity introduces several security challenges, including impersonation attacks. In such an attack, an adversary uses a stolen or fabricated identity to masquerade as another vehicle [45], effectively spoofing a legitimate neighbor. Once this spoofed link is established, the attacker can inject malicious data, intercept and monitor messages exchanged within the V2V network, and log sensitive information for later misuse [46].

Another prominent threat in V2X environments is the Denial-of-Service (DoS) attack [47]. In a DoS scenario, an adversary overwhelms the communication channel with interference or superfluous traffic, preventing legitimate messages from being transmitted or received correctly. By saturating the channel or overloading network nodes with useless data, the attacker can disrupt normal operation, deny authorized users access to network services, and block safety-critical messages from reaching their intended destinations. Replay attacks constitute another threat in V2X networks. In such attacks, an adversary captures legitimate packets and later retransmits them, often at the network or transport layer, causing outdated but valid messages to be accepted again [48]. This can confuse traffic management and cooperative driving functions, mislead nearby vehicles, and ultimately jeopardize transportation safety.

The V2X communication landscape has evolved substantially beyond the DSRC-based model that dominated earlier intelligent transportation system research. Dedicated Short-Range Communications (DSRCs), based on IEEE 802.11p-derived technology, remains relevant from a historical and legacy-deployment perspective; however, Cellular Vehicle-to-Everything (C-V2X) has become a major technology for contemporary connected-vehicle communications. C-V2X supports direct communication among vehicles, infrastructure, and vulnerable road users, including V2V, V2I, and V2P communication, without requiring a cellular base station for the direct communication link. It can additionally support vehicle-to-network (V2N) communication through cellular networks [49].

This transition is particularly significant in the United States. The Federal Communications Commission (FCC) has transitioned Intelligent Transportation System (ITS) operations in the upper 30 MHz of the 5.9 GHz band, from 5.895 to 5.925 GHz, from DSRC toward C-V2X. The FCC subsequently finalized the technical rules governing C-V2X operation in this spectrum, with the final rules becoming effective in 2025 [49,50].

The evolution of C-V2X also extends beyond its initial LTE-based implementations. 3GPP specifications address V2X communication based on both LTE and 5G New Radio (NR), reflecting the continuing evolution of cellular vehicular communication [51]. From a cybersecurity perspective, this technological transition does not eliminate established threats such as spoofing, replay, impersonation, denial-of-service, and message manipulation. Instead, it broadens the communication architecture and introduces additional dependencies on cellular infrastructure, roadside units, network services, and external communication interfaces. Consequently, cybersecurity-oriented HIL/VIL environments should represent the V2X technology and deployment model relevant to the vehicle under test rather than assuming a DSRC-only communication architecture.

#### 2.2.3. Physical-Layer Attacks

Modern vehicles increasingly incorporate a wide range of advanced sensors—such as LiDAR, millimeter-wave radar, cameras, ultrasonic sensors, and GPS—to support environment perception and autonomous decision-making. They also include numerous ECUs and wireless interfaces to enable intelligent connectivity. Although these technologies improve vehicle safety, automation, and efficiency, they also introduce new attack surfaces at the physical layer [14].

These physical-layer attacks can be executed through direct access, where an adversary might exploit the OBD-II port [52], tamper with sensors, reflash ECU firmware, or install malicious hardware like a CAN injector. However, physical contact is not always necessary. For instance, Rouf et al. [53] demonstrated that the Tire Pressure Monitoring System (TPMS) can be disrupted by injecting malicious radio-frequency signals, rendering the monitoring system ineffective. Similarly, Yang et al. [54] showed that keyless entry and start systems can be attacked by manipulating their radio communication channels, allowing adversaries to unlock and start a vehicle illegally.

#### 2.2.4. Remote Attacks

One major category of threats involves remote attacks that exploit wireless communication interfaces such as Wi-Fi [55,56,57], Bluetooth [58,59], satellite communication, and cellular networks. By leveraging vulnerabilities in these channels, adversaries can execute remote exploits and gain unauthorized access or control over critical vehicle functions.

A well-known example is the 2016 remote attack on a Tesla Model S demonstrated by Tencent Keen Security Lab. The researchers reported a remote, non-physical attack chain against an unmodified vehicle and demonstrated that vulnerabilities in connected vehicle components could enable unauthorized control of selected vehicle functions while the vehicle was parked or driving [21].

#### 2.2.5. Cloud, Backend, API, and Connected-Service Attacks

The attack surface of contemporary connected and software-defined vehicles extends beyond the vehicle itself to cloud infrastructure, manufacturer backend systems, mobile applications, web services, and application programming interfaces (APIs). These off-board components support functions such as remote vehicle access, telemetry, account management, diagnostics, software updates, and connected services. Consequently, vulnerabilities in backend infrastructure or external services can provide attackers with a remote path to vehicle-related data and functions without requiring direct access to the in-vehicle network. Recent automotive threat analyses identify cloud infrastructure and third-party integrations as increasingly important attack surfaces as vehicle-to-cloud connectivity expands [14].

Weak authentication, authorization, or access-control mechanisms in connected-vehicle APIs can be particularly significant because a single backend service may interact with a large population of vehicles. A notable example was disclosed in 2024 by Curry et al., who identified vulnerabilities in Kia’s connected-vehicle infrastructure that allowed unauthorized remote commands to be issued to affected vehicles using information associated with the vehicle license plate. The researchers also demonstrated unauthorized access to vehicle-owner information and the ability to add an additional account to a vehicle [60]. This type of attack differs fundamentally from traditional CAN injection because the initial compromise occurs through an off-board service, while its consequences can ultimately propagate to vehicle functions.

This evolution has direct implications for cybersecurity-oriented HIL/VIL validation. Test environments intended to represent modern connected vehicles should therefore consider not only local ECU and network attacks but also the effects of compromised telematics interfaces, backend commands, API requests, and cloud-to-vehicle communication. Depending on the scope of the testbed, these external services may be represented through emulated interfaces or controlled communication gateways so that their downstream effects on ECUs, network traffic, control signals, and vehicle behavior can be observed. Such cross-layer experimentation is increasingly important as automotive attacks span off-board infrastructure and in-vehicle systems rather than remaining confined to a single communication bus.

#### 2.2.6. Firmware, OTA, and Software-Supply-Chain Attacks

The increasing software content and connectivity of contemporary vehicles have expanded the automotive attack surface beyond individual ECU firmware. Modern vehicles depend on remotely updateable software, backend update infrastructure, third-party software components, and software supplied by multiple organizations across the automotive supply chain. Consequently, cybersecurity weaknesses may arise not only from vulnerabilities in the software executing on an ECU but also from the mechanisms used to develop, distribute, authenticate, and install that software [61,62].

Over-the-Air (OTA) software updates are particularly important because they enable manufacturers to deploy security patches and functional improvements without physical access to the vehicle. However, the update mechanism itself constitutes a security-sensitive communication path. Potential threats include unauthorized firmware modification, installation of malicious or untrusted software, compromise of update servers, man-in-the-middle attacks against the distribution channel, exploitation of update-protocol vulnerabilities, and firmware rollback attacks in which an older and more vulnerable software version is installed on a target ECU [61,62]. NHTSA therefore recommends protecting the integrity of OTA update servers, transmission mechanisms, and update processes, as well as restricting firmware modification to appropriately authenticated and authorized entities [62].

Software-supply-chain dependencies further broaden this threat model. Automotive manufacturers integrate software and electronic components from multiple suppliers, including commercial off-the-shelf and open-source software. A vulnerability introduced in a shared software component may therefore affect multiple ECUs, vehicle models, or product generations. NHTSA recommends that manufacturers and suppliers maintain inventories of hardware and software components and sufficient software-component information to identify affected ECUs and vehicles when new vulnerabilities are discovered [62]. These dependencies are particularly relevant to software-defined vehicles, where software services and updates continue to evolve throughout the operational lifetime of the vehicle.

From a cybersecurity-validation perspective, these developments imply that HIL/VIL environments should not restrict software-oriented testing to direct ECU reflashing or local firmware manipulation. Representative experiments can also reproduce compromised or unauthorized update conditions, version rollback, malformed update data, or manipulated communication between update infrastructure and vehicle components while observing the resulting ECU, network, and cyber–physical behavior. Such testing provides a controlled means of determining whether software-integrity mechanisms, gateways, and intrusion detection functions prevent a compromised software path from propagating toward safety-relevant vehicle functions [10,62].

#### 2.2.7. Sensor Spoofing and Perception Attacks

Modern vehicles increasingly rely on heterogeneous sensing systems, including cameras, LiDAR, radar, ultrasonic sensors, Global Navigation Satellite System (GNSS) receivers, and wheel-speed or other control-related sensors, to support localization, environment perception, driver-assistance functions, and closed-loop vehicle control. This dependence creates an important cyber–physical attack surface because an adversary may manipulate either the physical signal received by a sensor or the digital information subsequently communicated to an ECU. Unlike conventional network attacks, successful sensor and perception attacks can cause a controller to act on physically plausible but incorrect information, making them particularly difficult to distinguish from legitimate environmental changes or sensor faults [27,63,64].

GNSS spoofing is a representative example. By transmitting counterfeit or manipulated navigation signals, an attacker can cause a receiver to estimate an incorrect position or trajectory, potentially leading to navigation errors or unsafe automated-driving decisions [65]. Abrar et al. [66] investigated anomaly-based GPS spoofing detection using vehicle-behavior information, illustrating that the physical consistency between reported position and vehicle motion can provide useful evidence for identifying such attacks. This type of cross-layer analysis is particularly relevant to automotive cybersecurity because the attack may appear plausible at the sensor interface while remaining inconsistent with other physical or control-system variables.

Other perception modalities are also vulnerable to intentional manipulation. Contactless physical attacks can interfere with sensor measurements without requiring direct access to the in-vehicle network. Shoukry et al. [67], for example, demonstrated non-invasive spoofing of anti-lock braking system sensing, showing that malicious physical signals can alter measurements used by a safety-critical controller. Similar threat principles apply to camera-, radar-, LiDAR-, and ultrasonic-based perception, where manipulated, suppressed, or fabricated observations may cause false-object detection, missed obstacles, incorrect ranging, or degraded environmental awareness [63,64].

A distinct class of perception attack targets the physical environment rather than directly manipulating the electrical or communication signal received by a sensor. Eykholt et al. [68] demonstrated robust physical-world adversarial attacks in which carefully designed perturbations applied to road signs caused deep-learning-based visual classifiers to misclassify otherwise legitimate physical objects under different viewing conditions. Such attacks differ from conventional sensor spoofing because the sensing hardware may operate normally while an adversarially modified object in the environment induces an incorrect perception result.

Sensor attacks are therefore especially important for HIL-/VIL-based cybersecurity validation. A controlled test environment can substitute, perturb, delay, replay, or otherwise manipulate sensor inputs while preserving closed-loop vehicle dynamics and real ECU execution. The resulting effects can then be observed simultaneously through sensor measurements, controller states, actuator commands, network traffic, and vehicle-dynamic variables. Such experiments enable researchers to distinguish nominal operation, conventional sensor faults, and malicious manipulation while also providing synchronized cyber–physical data for AI-based detection. Consequently, the evaluation of sensor spoofing should consider not only whether the manipulated measurement is detected, but also detection latency, false-alarm behavior, robustness across vehicle operating conditions, and whether detection occurs before the attack produces an unacceptable physical consequence.

Table 2 summarizes representative survey and review articles on automotive cybersecurity published between 2019 and 2026. The table highlights how the literature organizes threats across major attack surfaces—including in-vehicle networks, remote and firmware-based vectors, and sensor/perception manipulation—and, where applicable, categorizes corresponding defense strategies such as security architectures, authentication mechanisms, and AI-based intrusion detection. Overall, these surveys collectively emphasize that modern vehicle security is inherently cyber–physical: attacks can influence not only data integrity and availability but also control behavior and safety outcomes.

These attacks can trigger incorrect perception, unsafe control actions, or destabilization of autonomous driving algorithms. Consequently, Automotive Cybersecurity has emerged as a critical field focused on developing comprehensive measures to protect vehicles. This includes designing security architectures to prevent attacks, implementing detection mechanisms to identify ongoing intrusions, and creating reactive countermeasures to contain and respond to malicious activities. In light of these demonstrated risks, modern ECUs must now operate under increasingly stringent cybersecurity requirements to ensure system integrity, data authenticity, and operational safety. Therefore, it is necessary to perform cybersecurity tests in vehicles to reveal and address relevant security threats and vulnerabilities.

### 2.3. Automotive Cybersecurity Standards and Regulatory Context

The automotive cybersecurity landscape changed significantly with the introduction of dedicated international engineering standards and vehicle regulations. ISO/SAE 21434:2021, Road Vehicles—Cybersecurity Engineering, establishes a lifecycle-oriented framework for managing cybersecurity risks associated with the electrical and electronic systems of road vehicles. Its scope extends across concept, product development, production, operation, maintenance, and decommissioning, thereby integrating cybersecurity activities into the broader automotive engineering lifecycle [72]. The standard provides a systematic basis for identifying cybersecurity risks, defining cybersecurity requirements, implementing appropriate controls, and maintaining cybersecurity throughout the vehicle lifecycle.

At the regulatory level, UN Regulation No. 155 establishes requirements concerning vehicle cybersecurity and the Cyber Security Management System (CSMS). It provides a framework for vehicle type approval with respect to cybersecurity and requires manufacturers to establish organizational processes for identifying, assessing, mitigating, and managing cybersecurity risks throughout the relevant vehicle lifecycle [73]. Consequently, cybersecurity is no longer solely an engineering best practice but is also connected to regulatory approval and organizational cybersecurity-management processes in markets applying the UNECE vehicle-regulation framework.

UN Regulation No. 156 complements this framework by addressing software updates and the Software Update Management System (SUMS). It establishes requirements for managing software versions and update processes, including the identification of software relevant to vehicle type approval and the protection, verification, and validation of software-update mechanisms [74]. These requirements are increasingly important for software-defined and connected vehicles, in which functionality and cybersecurity protections may continue to evolve through OTA updates after vehicle production.

These standards and regulations also provide important context for cybersecurity-oriented HIL/VIL validation. ISO/SAE 21434 and UN Regulations Nos. 155 and 156 do not prescribe HIL or VIL as mandatory test methodologies. However, controlled HIL/VIL experimentation can support cybersecurity verification and validation activities by enabling repeatable evaluation of attack scenarios, security controls, software-update behavior, network segmentation, intrusion-detection mechanisms, and their cyber–physical consequences. In this sense, HIL/VIL platforms can provide experimental evidence supporting engineering assurance activities while preserving safe and reproducible conditions for potentially disruptive cybersecurity tests.

Assessing the impact of cyberattacks on modern vehicles requires a robust testing platform to evaluate their effects on real vehicle systems. HIL simulation is ideally suited for this task. Already a critical tool in the automotive development cycle, HIL testing accelerates product validation and complements physical vehicle tests, providing a controlled environment to precisely detect, quantify, and analyze the consequences of cyberthreats.

Taken together, the surveyed literature illustrates that evaluating automotive cyberthreats requires more than software-only analysis: realistic testing must capture timing constraints, network behavior, and closed-loop control dynamics while maintaining safety and repeatability. As a result, controlled experimental platforms are essential for quantifying the cyber–physical impact of attacks on safety-critical ECUs and for benchmarking detection and mitigation strategies under reproducible conditions. This motivates the use of HIL simulation, which enables real ECU hardware to be exercised within real-time virtual vehicle and environment models, bridging the gap between pure simulation and costly, high-risk full-vehicle experimentation. The next section therefore discusses the role of HIL simulation as a foundation for automotive cybersecurity validation.

## 3. Cybersecurity-Oriented HIL/VIL and Digital-Twin Validation

Evaluating automotive cybersecurity requires experimental platforms capable of reproducing not only malicious network or software activity but also the resulting effects on physical vehicle behavior. Purely software-based cybersecurity analysis can identify protocol weaknesses, malformed traffic, or vulnerable interfaces, but it may not fully capture how an attack propagates through real ECU timing, sensor–controller interactions, actuator commands, and closed-loop vehicle dynamics [10,75]. Conversely, intentionally executing potentially unsafe attacks on complete vehicles under road conditions is costly, difficult to reproduce, and may introduce unacceptable safety risks [10,75].

HIL simulation provides an intermediate validation environment in which real automotive hardware, such as an ECU or controller, interacts in real time with simulated vehicle dynamics, sensors, actuators, and environmental conditions. This configuration enables cybersecurity experiments to be conducted while preserving realistic ECU execution, communication timing, and closed-loop control behavior. Attack scenarios can therefore be introduced under controlled and repeatable conditions, and their effects can be observed simultaneously in the cyber domain, through network and ECU activity, and in the physical domain, through changes in sensor signals, actuator commands, control performance, and simulated vehicle dynamics [75,76,77,78,79].

VIL extends this principle by incorporating a physical vehicle, or a larger portion of the vehicle, into a controlled virtual environment. VIL configurations can therefore capture additional interactions among vehicle hardware, communication systems, dynamics, and, in some cases, human behavior that may not be represented in ECU-level HIL experiments [75,77]. From a cybersecurity perspective, HIL and VIL should consequently be viewed not merely as functional verification platforms, but as controlled cyber–physical experimentation environments for reproducing attacks, measuring their consequences, generating representative security data, and evaluating detection and mitigation mechanisms.

Despite these advantages, cybersecurity-oriented HIL testing presents several implementation challenges. Real-time vehicle models must execute deterministically, physical and network interfaces must preserve relevant timing characteristics, and the fidelity of simulated sensors, actuators, communication channels, and vehicle dynamics must be sufficient for the attack under investigation [76,78,79]. These requirements become more demanding as cybersecurity experiments expand from individual ECUs or CAN segments toward heterogeneous vehicle networks, multiple controllers, connected services, and VIL configurations. Nevertheless, compared with uncontrolled road testing, HIL/VIL environments provide substantially greater repeatability, observability, and safety for evaluating potentially disruptive or hazardous cyberattack scenarios [10,75,77].

A further limitation is that laboratory HIL/VIL environments cannot necessarily reproduce all physical and environmental conditions experienced by a production vehicle. Factors such as temperature extremes and thermal cycling, vibration and mechanical stress, electromagnetic disturbances, and differences between laboratory interfaces and actual vehicle installations may influence ECU behavior, sensor performance, and communication reliability [80,81,82,83]. Unless dedicated environmental, electromagnetic-compatibility, or mechanical test equipment is incorporated into the platform, such effects may be simplified or omitted from conventional HIL/VIL experiments. Consequently, HIL/VIL testing should be viewed as complementary to environmental, EMC, proving-ground, and full-vehicle validation rather than as a complete replacement for these forms of testing [75,80].

### 3.1. HIL and VIL for Automotive Cybersecurity Testing

A cybersecurity-oriented HIL environment generally combines three functional domains: the physical automotive hardware under test, a real-time representation of the vehicle and its operating environment, and an attack or network-manipulation interface. The hardware under test may consist of an individual ECU, several interconnected controllers, a gateway, communication interfaces, telematics hardware, or a larger vehicle subsystem. The simulated portion provides the sensor inputs, actuator loads, vehicle dynamics, and environmental conditions required for closed-loop operation. A separate attack interface can then manipulate network communication, sensor information, or other system inputs while the test platform records both cyber and physical responses [76,77,79].

This architecture is particularly important for automotive cybersecurity because attacks that appear similar at the network level can produce substantially different physical consequences depending on the operating state of the vehicle. For example, injection or modification of a control-related message may have little effect under one operating condition but may alter braking, steering, propulsion, or stability behavior under another. Closed-loop HIL testing enables these dependencies to be examined systematically while maintaining controlled initial conditions and repeatable attack parameters [75,76,77].

Existing automotive cybersecurity platforms illustrate different levels of physical integration. Oruganti et al. [76] demonstrated an HIL-based automotive embedded-system cybersecurity testbed that combines vehicular communication and physical-system behavior. More recent HIL/VIL platforms have extended this concept toward broader cyber–physical experiments. Weaver et al. [75] emphasized VIL configurations for investigating cybersecurity effects that may be missed by software-only evaluation, while Kang and Jeon [77] coupled a physical vehicle with a virtual environment for repeatable real-time automotive cyberattack experiments. Cost-contained and hybrid platforms such as HackCar [84] provide another intermediate level between software simulation and full-vehicle experimentation.

These platforms therefore occupy different positions along a simulation-to-physical continuum. Software-only environments offer scalability and low experimental cost; ECU-level HIL introduces real controller execution and interfaces; subsystem-level HIL increases physical fidelity; and VIL incorporates a substantially larger portion of the actual vehicle. Selecting among these configurations should depend on the attack surface and cyber–physical phenomenon being evaluated rather than solely on the availability of hardware [10,75,77].

Table 3 summarizes representative automotive cybersecurity test environments spanning software simulation, hybrid platforms, HIL, and VIL configurations. The comparison illustrates the increasing degree of physical integration across these platforms and their respective roles in controlled cybersecurity experimentation.

### 3.2. Cyberattack Injection and Cyber–Physical Data Acquisition

The value of HIL/VIL for cybersecurity evaluation depends on the ability to reproduce an attack in a controlled manner and to observe the resulting behavior across both cyber and physical domains. Unlike conventional functional fault injection, cybersecurity experiments intentionally manipulate information, communication, or software behavior in ways that represent adversarial actions. Depending on the target architecture and attack surface, this manipulation can occur at the in-vehicle network, sensor, communication-interface, or controller level [10,76,77].

For in-vehicle network attacks, the test environment can introduce malicious or modified traffic while the real ECU continues to interact with the simulated vehicle. Representative attack scenarios include message injection, replay, flooding or denial-of-service behavior, masquerading, and modification of control-relevant information. HIL-based experimentation allows attack parameters such as timing, message frequency, payload modification, and attack duration to be varied systematically while maintaining controlled vehicle operating conditions [76,77]. This repeatability is particularly important when comparing detection approaches because variations in the driving scenario or ECU operating state can otherwise influence the observed attack effects.

Sensor-oriented experiments require a different form of attack representation. Rather than manipulating only network frames, the HIL environment can modify or substitute sensor information presented to the controller while the underlying vehicle-dynamics model continues to operate in closed loop. This enables controlled evaluation of discrepancies between expected physical behavior and compromised measurements. Fault-injection frameworks provide useful mechanisms for generating such abnormal sensor and actuator conditions, although faults and intentional cyberattacks should be distinguished when interpreting the resulting data [12,79].

A major advantage of cybersecurity-oriented HIL/VIL experimentation is the ability to collect synchronized observations from multiple system layers. Depending on the configuration of the testbed, these observations may include network identifiers and payloads, message timing and frequency, ECU inputs and outputs, diagnostic variables, sensor measurements, actuator commands, controller states, and vehicle-dynamic variables [75,77,79]. Consequently, the same experiment can provide both cyber indicators and physical-response variables, enabling security mechanisms to move beyond network-only analysis toward cyber–physical behavior modeling.

For AI-based evaluation, the experimental configuration should additionally record contextual information describing the scenario, including vehicle operating state, attack onset and duration, attack parameters, affected components or communication channels, and the distinction among nominal, faulty, and malicious conditions. Such synchronization and labeling enable HIL/VIL experiments to support not only attack-impact analysis but also the generation and validation of datasets for intrusion and anomaly detection.

### 3.3. HIL/VIL-Generated Data for AI-Based Detection

Beyond attack-impact evaluation, HIL/VIL environments can provide an important source of controlled cyber–physical data for the development and evaluation of AI-based security mechanisms. Because the experimenter controls the operating scenario, attack parameters, and system configuration, nominal and abnormal conditions can be reproduced while synchronized measurements are collected from network, ECU, sensor, actuator, and vehicle-dynamic layers [75,76,77,79]. This capability is particularly valuable in automotive cybersecurity, where obtaining accurately labeled attack data from production vehicles is difficult and intentionally executing safety-critical attacks on public roads is generally impractical [10,75].

The resulting datasets may contain several complementary forms of information. Network-level observations can include message identifiers, payloads, transmission intervals, frame frequency, and other communication characteristics. ECU- and control-level observations may include controller inputs and outputs, diagnostic variables when accessible, and actuator commands. Physical-level observations can include sensor measurements and vehicle-dynamic variables generated by the underlying HIL/VIL model. Combining these data sources enables security mechanisms to identify not only anomalous communication patterns but also inconsistencies between network activity and expected cyber–physical behavior [76,77,79].

HIL/VIL-generated data can support different classes of AI-based detection. Supervised approaches can be trained when representative abnormal or attack scenarios are explicitly generated and labeled; for example, Abboush et al. [88] used an HIL-based real-time fault-injection framework to generate automotive signal data for training, validation, and testing of a deep-learning-based detection and classification model. When abnormal examples are scarce, normal-only anomaly-detection approaches can instead learn nominal temporal behavior and identify deviations from the learned pattern [89]. Temporal models are particularly relevant to automotive cybersecurity because sequential dependencies in communication and control signals can provide discriminative information; for instance, Desta et al. [90] used an LSTM to model sequences of CAN arbitration identifiers and detect deviations from expected message-order patterns.

More importantly, the controlled nature of HIL/VIL experimentation enables systematic variation of operating and attack conditions. Kang and Jeon [77] demonstrated a VILS framework in which attack type, target, timing, duration, and message-injection parameters can be configured and repeated under controlled driving conditions. Their repeated experiments further show that identical or systematically varied attack scenarios can be used to assess the consistency of cyber–physical responses. Such capabilities provide a basis for evaluating robustness and generalization across multiple operating and attack conditions rather than reporting detection performance for only a single fixed scenario.

The relationship between HIL-generated data and AI evaluation should nevertheless be interpreted carefully. A high-fidelity HIL environment does not automatically guarantee that the generated data fully represent attacks encountered in production vehicles, and high classification accuracy alone does not ensure suitability for real-time automotive deployment. Evaluation should therefore consider not only conventional detection metrics but also false-alarm behavior, detection latency, robustness across operating conditions, and, where applicable, whether an attack is detected before it causes an unacceptable cyber–physical consequence. HIL/VIL thus provides the experimental foundation for generating and validating realistic data, while Section 4 examines the AI-based methods used to interpret these data and identify malicious or abnormal behavior.

### 3.4. Digital Twins as a Bridge Between HIL and AI

Digital twins (DTs) provide a complementary mechanism for connecting physical automotive systems, virtual representations, experimental data, and data-driven security analysis. Although digital twins and HIL simulation both involve virtual system models, the two concepts should not be treated as synonymous. HIL primarily emphasizes real-time interaction between physical hardware and a simulated plant or environment, whereas a digital twin maintains a virtual representation that is linked to information from its corresponding physical system and can be used for monitoring, analysis, and decision support. This distinction is important because not every HIL configuration constitutes a digital twin, although HIL can provide physical interfaces and real-time data that support a digital-twin implementation [91,92].

In automotive cybersecurity, digital twins can extend conventional HIL/VIL experimentation by maintaining virtual representations of vehicle components, communication networks, and their interactions. Kabir and Ray [92], for example, developed the ViSE digital-twin platform for automotive functional-safety and cybersecurity exploration, enabling interactions among ECUs, sensors, actuators, and representative safety and security scenarios. Similarly, Sharmin et al. [93] proposed a digital-twin framework that reproduces vehicle ECU and CAN-bus behavior while incorporating real-time data from the physical network. Their framework is designed to generate realistic combinations of attack traffic and driving scenarios for evaluating the detection capability and performance of CAN intrusion-detection systems.

Digital twins can also provide an explicit bridge between cyber–physical experimentation and AI-based security mechanisms. Rather than using only a fixed offline dataset, a DT-enabled environment can provide contextual and dynamically generated observations associated with the state of the represented vehicle or communication system. Yigit et al. [94] demonstrated this concept in the Cyber-Twin framework, which combines digital-twin technology with AI-based attack detection for vehicular ad hoc networks and uses the twin for real-time monitoring and detection of attacks affecting roadside infrastructure. More recently, Wang et al. [95] constructed a digital twin of an Internet-of-Vehicles environment and generated multiple intrusion scenarios within the twin while applying an ensemble-learning-based intrusion detector. These studies illustrate how digital twins can connect controlled cyberattack representation, synchronized system observations, and data-driven detection within a unified experimental workflow.

Nevertheless, introducing a digital twin does not by itself guarantee a trustworthy cybersecurity evaluation. The usefulness of the twin depends on the fidelity of the underlying models, the integrity and synchronization of physical-to-virtual data, and the extent to which the represented operating and attack conditions reflect the real system. A recent systematic review of automotive digital-twin security identifies continuing challenges related to scalability, trust, safety, threat-model validation, and the security of the twin architecture itself [96]. More generally, cyber–physical resilience research has highlighted physical-model manipulation, semantic inconsistencies between models and deployed implementations, and limited cross-layer observability as persistent sources of vulnerability [97]. Consequently, digital twins should be considered a complementary layer to HIL/VIL rather than a replacement for physical cybersecurity validation. Their primary value in the present context is to strengthen the connection between repeatable cyber–physical experiments, continuously interpretable system representations, and AI-based detection and evaluation.

## 4. AI-Powered Cyber Threat Detection and Mitigation

As modern vehicles become increasingly connected and software-defined, automotive cybersecurity must move beyond rule-based and signature-based defenses that struggle to generalize to unseen attacks and evolving system behavior. The availability of realistic, time-synchronized sensor, actuator, and in-vehicle network traces—often obtained through controlled simulation and HIL/vehicle-in-the-loop experimentation—has accelerated the use of AI for data-driven detection and mitigation of cyberthreats targeting vehicles. In this setting, AI models learn normal cyber–physical behavior from sequences of signals and messages and flag deviations that indicate spoofing, replay, denial-of-service, or more subtle manipulation of control-relevant data. For example, Olufowobi et al. [98] proposed a specification-based intrusion detection system that takes advantage of supervised anomaly detection using a real-time system model as input. Similarly, Desta et al. [90] introduced a CAN intrusion detection approach based on sequence modeling: a trained Long Short-Term Memory (LSTM) network predicts the next arbitration identifier from the previous twenty IDs, and anomalies are detected when the observed identifier deviates from the prediction.

### 4.1. Foundational Machine Learning Approaches

Foundational machine learning methods remain widely used for automotive intrusion and anomaly detection because they can operate on features extracted from in-vehicle network traffic, ECU behavior, and vehicle-related signals. Automotive cybersecurity studies commonly formulate detection as supervised classification when labeled attack data are available, or as unsupervised or semi-supervised anomaly detection when representative attack labels are scarce [17,33,71]. Supervised methods such as Support Vector Machines (SVMs), Random Forests, Gradient Boosting, Naive Bayes, and decision trees have been applied to distinguish legitimate traffic from known attack classes such as spoofing, replay, and denial-of-service attacks. Unsupervised approaches, including clustering, Isolation Forests, and principal-component-based methods, instead seek deviations from learned nominal behavior and are therefore particularly relevant when previously unseen attacks must be considered.

Automotive studies have applied these conventional machine learning approaches directly to in-vehicle communication and connected-vehicle security. Derhab et al. [31], for example, proposed a histogram-based intrusion-detection and filtering framework for in-vehicle networks, illustrating how statistical characteristics of CAN traffic can be used to distinguish malicious from legitimate communication. Machine-learning approaches have also been investigated for connected vehicle infrastructure; Girdhar et al. [99] examined ML-based prediction and mitigation of cyberattacks targeting electric-vehicle charging stations. These studies illustrate that conventional ML remains relevant to automotive cybersecurity when suitable features can be extracted from network traffic or system behavior, although its effectiveness depends strongly on the representativeness of the training data and the operating conditions under which the detector is evaluated.

By modeling normal ECU behavior and flagging deviations, unsupervised methods provide adaptable anomaly detection capabilities that are particularly suited to emerging or zero-day threats. Despite these advantages, traditional ML models often struggle to capture highly dynamic time-series patterns and complex nonlinear behaviors present in automotive systems.

### 4.2. Deep Learning for Complex Pattern Recognition

Within machine learning, deep learning (DL) methods have become increasingly important for automotive intrusion and anomaly detection because they can learn complex spatial and temporal relationships directly from in-vehicle network, ECU, sensor, and cyber–physical data [71,88]. Convolutional Neural Networks (CNNs) can extract discriminative patterns from multidimensional automotive signal representations, whereas recurrent architectures such as Long Short-Term Memory (LSTM) networks are well suited to sequential vehicle data in which message order, timing, and temporal dependencies are important for distinguishing nominal behavior from malicious activity [90,100].

Temporal modeling is particularly relevant to automotive cybersecurity because many attacks alter not only individual message values but also the expected sequence or timing characteristics of vehicle communication. Desta et al. [90], for example, used an LSTM-based model to learn sequences of CAN arbitration identifiers and detect deviations from expected message-order behavior. Thiruloga et al. [100] proposed TENET, a temporal convolutional neural network with attention for anomaly detection in automotive cyber–physical systems. The use of temporal convolutions and attention mechanisms enables the detector to capture longer-range dependencies while emphasizing signal or temporal features that are most informative for identifying abnormal behavior.

More recent work has continued this trend toward attention-based automotive intrusion detection. Saravanan et al. [101] developed an attention-based deep learning intrusion-detection and classification model for CAN messages, illustrating the increasing use of attention mechanisms to identify relevant relationships within automotive network traffic. Such approaches are particularly attractive for modern in-vehicle networks because they can model complex traffic patterns without requiring all attack signatures to be defined explicitly in advance.

Deep learning models have also been integrated with automotive HIL-based experimentation. Abboush et al. [88] used a hybrid CNN–LSTM architecture with data generated through an automotive HIL-based fault-injection environment to detect and classify abnormal sensor and actuator behavior. Although fault injection is not equivalent to intentional cyberattack injection, this study demonstrates how a controlled HIL platform can generate synchronized abnormal cyber–physical data for developing and evaluating data-driven detection models. More recent HIL-oriented cybersecurity frameworks further illustrate how real-time experimental platforms can support the validation of automotive intrusion-detection strategies under repeatable network and operating conditions [102].

These studies demonstrate that the value of deep learning for automotive cybersecurity depends not only on classification accuracy but also on the relationship among the selected vehicle signals, attack mechanism, temporal behavior, and experimental platform. For HIL/VIL-based validation, deep-learning models should therefore be evaluated using representative attack conditions and realistic communication timing, with attention to false-alarm behavior, detection latency, and robustness across different vehicle operating states.

### 4.3. Emerging AI Architectures and Deployment Considerations

Recent automotive intrusion-detection research is expanding beyond conventional CNN, RNN, and transformer architectures toward models that more explicitly address long-sequence efficiency, relational structure, generative learning, and resource-constrained deployment. State-space models are one emerging direction because they can model long temporal sequences with computational characteristics that differ from recurrent and full self-attention mechanisms. Feng et al. [103], for example, proposed MambaCNN for Internet-of-Vehicles intrusion detection by combining a selective state-space model with convolutional feature extraction. Their framework considers both individual CAN messages and message sequences and further applies knowledge distillation to reduce model complexity. Such approaches are relevant to automotive IDS design because long communication traces must often be processed under strict latency and memory constraints.

Graph-based methods provide a complementary direction by representing relationships among CAN identifiers, signals, or communication events rather than treating each message only as an independent feature vector. Song et al. [104] proposed a dynamic graph-based IDS in which CAN graphs are updated as messages arrive, enabling real-time anomaly detection and identification of the attacked message identifier. More recently, Gao et al. [105] modeled dependencies among reverse-engineered CAN signals using graph neural networks and graph transformers. These methods illustrate how structural and relational information can complement temporal models when attacks disturb dependencies among otherwise individually plausible vehicle signals or messages.

Generative approaches are also increasingly relevant to automotive intrusion detection, particularly when labeled attack data are limited or when unknown attacks must be considered. Wang et al. [106] developed a dual-discriminator GAN-based IDS for CAN-FD traffic and used the generative framework to distinguish normal and anomalous ID patterns, including previously unseen attacks. Rangsikunpum et al. [107] combined a two-stage binarised neural network with a GAN to support both unknown-attack detection and known-attack classification. These studies show that generative models can contribute not only to synthetic data augmentation but also to distribution learning and novelty detection for in-vehicle networks.

Model architecture alone, however, is insufficient for practical automotive deployment. Intrusion detectors must satisfy constraints on inference latency, throughput, memory, power consumption, hardware resources, and coexistence with safety-critical ECU workloads. Khandelwal and Shanker [108] demonstrated a quantised CAN IDS mapped to the Xilinx Deep Learning Processing Unit (DPU) IP on a Zynq UltraScale+ FPGA, reducing per-message latency and power relative to GPU-oriented implementations. Rangsikunpum et al. [107] similarly demonstrated sub-millisecond inference on a low-cost FPGA. Consequently, evaluation of emerging automotive IDS architectures should report not only accuracy, precision, recall, and F1-score, but also detection latency, false-alarm behavior, memory and logic utilization, power or energy consumption, and robustness across vehicles, operating conditions, and attack distributions. These deployment-oriented metrics are particularly important when AI-based security functions are intended to operate online within ECUs, gateways, or HIL/VIL validation platforms.

### 4.4. Automotive Cybersecurity Datasets and Benchmarking

Publicly available automotive cybersecurity datasets play an important role in the development and comparison of AI-based intrusion detection systems. However, the characteristics of these datasets vary substantially in terms of vehicle platform, attack type, signal representation, data-collection conditions, and whether attacks are physically executed or synthetically generated. Consequently, high detection performance obtained on one dataset does not necessarily imply equivalent performance on another vehicle, network architecture, or operating condition.

One of the most widely used automotive intrusion-detection resources is the HCRL Car-Hacking Dataset, which contains CAN traffic collected from a real vehicle under normal operation and several message-injection attacks, including denial-of-service, fuzzing, and gear- and RPM-spoofing scenarios [109]. Because individual injected messages are labeled, the dataset is particularly suitable for supervised classification and has been used extensively for evaluating AI-based CAN intrusion detectors. However, its attack set primarily represents relatively direct CAN injection scenarios and therefore does not fully capture the diversity of attacks found in contemporary heterogeneous and connected vehicle architectures.

The Real ORNL Automotive Dynamometer (ROAD) dataset was introduced to address several limitations of earlier CAN IDS datasets [110]. ROAD contains more than 3.5 h of real automotive CAN traffic and includes ambient driving data together with fuzzing, fabrication, advanced attacks, and simulated masquerade attacks. It also provides signal-translated time-series representations for a subset of the CAN captures. Importantly, the associated analysis emphasizes that many public CAN datasets contain easily detectable injection attacks, limited vehicle diversity, or insufficient information about physical attack consequences. These limitations can make direct comparison among IDS studies difficult and may overestimate the ability of a model to generalize to realistic attacks.

Recent dataset-development efforts have consequently placed greater emphasis on cross-vehicle evaluation, more diverse attack classes, and the ability to test generalization to previously unseen attacks. Such considerations are especially important for AI-based automotive IDSs because model performance can depend strongly on the distribution of vehicle-specific CAN identifiers, payload encodings, message timing, operating states, and attack-generation procedures. Evaluation should therefore distinguish between training and testing on the same vehicle and attack distribution and more demanding experiments involving unseen attacks, different operating conditions, or different vehicle platforms.

HIL-/VIL-generated datasets provide a complementary approach to public benchmark data. Unlike fixed offline datasets, controlled HIL/VIL environments can vary vehicle operating state, attack onset, duration, severity, and affected signals while collecting synchronized network and physical measurements. Public benchmark datasets are therefore valuable for reproducibility and comparison, whereas HIL-/VIL-generated data can support controlled evaluation of cyber–physical effects, timing constraints, and generalization across operating conditions. A robust automotive AI evaluation methodology should ideally combine both forms of evidence rather than relying exclusively on classification accuracy obtained from a single offline dataset.

### 4.5. Cyber–Physical Behavior Modeling and HIL-Based Detection

Automotive intrusion and anomaly detection can benefit from models that consider relationships among network communication, ECU behavior, sensor measurements, and physical vehicle dynamics rather than examining individual signals in isolation. Such cyber–physical behavior modeling is particularly relevant when an attack produces only subtle changes in network traffic but causes measurable inconsistencies between communicated information and the expected physical or control-system response. Sequence- and specification-based approaches provide examples of this principle. Olufowobi et al. [98] used CAN timing and system specifications for automotive intrusion detection, while Desta et al. [90] modeled CAN identifier sequences to identify deviations from expected communication behavior.

HIL/VIL platforms extend this concept by providing synchronized observations from both cyber and physical layers under controlled abnormal conditions. Abboush et al. [88], for example, generated automotive sensor and actuator data using an HIL-based fault-injection environment and used a CNN–LSTM model for detection and classification. For vehicle-positioning attacks, Abrar et al. [66] investigated anomaly-based detection of GPS spoofing using vehicle-behavior information. These examples illustrate how detection can incorporate control-relevant or physical information in addition to network characteristics.

Real-time implementation is also important when AI-based detection and cyber–physical analysis are intended for embedded automotive systems. Li et al. [102] proposed an FPGA-based HIL framework for accelerating the validation of automotive network-security strategies.

In related work, Kordi et al. [111] developed an FPGA-based AI-driven HIL platform for real-time ABS ECU testing in which a Temporal Convolutional Network (TCN) was implemented as a virtual wheel-speed sensor. The platform demonstrated deterministic low-latency closed-loop operation and showed how AI inference can be integrated directly within an automotive HIL environment for real-time ECU validation. Although this study focused primarily on functional ECU testing rather than cyberattack detection, its architecture provides a relevant example of how AI models can be deployed within FPGA-based HIL platforms under real-time automotive constraints.

Kordi et al. [112] developed an FPGA-based dual-learning model for wheel-speed sensor anomaly detection in an ABS HIL environment. The proposed architecture was designed to distinguish nominal and abnormal sensor behavior while satisfying closed-loop real-time execution constraints. This study provides an example of how embedded AI inference can be incorporated into HIL-based cyber–physical anomaly detection.

### 4.6. Adversarial Robustness of AI-Based Automotive Intrusion Detection

Although AI-based intrusion detection systems can identify complex or previously unseen automotive attacks, the learning model itself introduces an additional attack surface. Adversarial machine learning (AML) attacks can target a detector either during inference or during model development. In an evasion attack, an adversary modifies malicious inputs at inference time so that they remain operationally effective while being classified as benign. In contrast, poisoning attacks manipulate training samples, labels, or model updates with the objective of degrading the decision boundary or creating systematic misclassification behavior. Consequently, high detection accuracy on an unmodified automotive dataset does not by itself demonstrate that an AI-based IDS is robust against an adaptive attacker [113,114,115].

Recent automotive studies demonstrate that such attacks are practically relevant to in-vehicle intrusion detection. Longari et al. [113] investigated adversarial evasion against CAN-based automotive IDSs while explicitly considering the time-dependent characteristics of automotive network traffic. Their later study [115] extended this analysis to white-box, gray-box, and black-box attacker-knowledge scenarios and examined the feasibility of generating evasive payloads for timed injection into CAN traffic. Aloraini et al. [114] similarly demonstrated that a surrogate IDS trained from accessible in-vehicle data can be used to construct black-box adversarial examples while respecting relevant IVN traffic constraints. Their experiments showed substantial degradation of victim IDS performance, illustrating that models exhibiting high nominal detection accuracy may remain vulnerable to adversarial manipulation.

More recent work confirms that this concern extends across both CAN and connected-vehicle environments. Mamun et al. [116] evaluated several adversarial attacks against AI-based IDS models using the CICIoV2024 V2X dataset and reported substantial degradation in detection performance. Barletta et al. [117] investigated Boundary and HopSkipJump attacks against supervised CAN-frame IDS models in a pure black-box setting, demonstrating that decision-based attacks can significantly reduce detection effectiveness even when the attacker lacks knowledge of the internal model. These results indicate that robustness should be evaluated not only against conventional spoofing, replay, injection, and denial-of-service traffic, but also against attack variants specifically optimized to avoid an AI-based detector.

Training-time manipulation represents a complementary threat. Pooranian et al. [118] investigated label-flipping data-poisoning attacks against deep-learning-based intrusion detection in connected vehicles. Such attacks are particularly relevant to IDS architectures that are periodically retrained, updated from newly collected vehicle data, or trained collaboratively, because an attacker who influences the training data can degrade the detector before deployment. Therefore, the provenance, integrity, and labeling of automotive cybersecurity datasets should be considered part of the security of the AI pipeline rather than only a data-preparation issue.

Several defenses have consequently been investigated. Lai et al. [119] proposed a gradient-correlation method for identifying adversarial samples in vehicular networks without requiring retraining of the original detection model. Lin et al. [120] evaluated adversarial training, ensemble learning, and distance-based optimization for improving the resilience of CAN-bus ML-based IDSs, while Barletta et al. [117] also demonstrated the usefulness of adversarial training for improving robustness against black-box manipulation. These findings suggest that automotive IDS evaluation should report adversarial robustness in addition to conventional accuracy, precision, recall, and F1-score.

From an HIL/VIL perspective, adversarial robustness represents an important extension of conventional AI validation. A controlled cyber–physical environment can be used to introduce adversarially modified network or sensor inputs while maintaining realistic communication timing, vehicle operating conditions, and closed-loop dynamics. Evaluation can then determine not only whether an adversarial example bypasses the IDS, but also whether the resulting undetected manipulation produces an unacceptable physical effect. HIL/VIL therefore provides a useful experimental layer for jointly evaluating classification robustness, detection latency, attack feasibility, and cyber–physical consequences under adversarial AI conditions.

Although research on automotive AI-based intrusion detection is extensive, comparatively fewer studies integrate AI-based detection with explicit simulation, HIL, or VIL-based cybersecurity validation. Table 4 therefore summarizes representative studies that explicitly connect these dimensions. The selected works cover diverse attack and anomaly scenarios, including CAN-bus intrusions, GNSS/GPS spoofing, automotive network attacks, and cyber–physical sensor anomalies, and illustrate how controlled test environments can support repeatable evaluation under realistic timing and closed-loop conditions.

## 5. Integrated AI–HIL Framework for Automotive Cybersecurity Evaluation

The preceding sections demonstrate that automotive cybersecurity, cyber–physical validation, and AI-based intrusion detection should not be considered independently. The effectiveness of an automotive IDS depends not only on the learning algorithm itself but also on whether the underlying test environment realistically represents the vehicle architecture, attack mechanism, timing behavior, physical dynamics, and signals affected by the attack. Conversely, an HIL or VIL platform provides limited cybersecurity insight if the generated attack scenarios and measured signals are not connected to appropriate detection and evaluation mechanisms.

The integration of AI with HIL/VIL can be understood as a two-stage experimental workflow. During model development, the HIL/VIL platform generates synchronized cyber–physical observations under nominal and controlled attack conditions, which can be used to train, validate, or benchmark AI-based detection models. During closed-loop evaluation, a trained detector can operate online using streaming network, ECU, sensor, or vehicle-state data obtained from the test environment. The resulting alarms can then be evaluated together with detection latency, false-alarm behavior, cyber–physical attack consequences, and, where implemented, mitigation or response actions.

Representative studies illustrate different parts of this integration. Abboush et al. [88] used HIL-generated automotive data to develop and evaluate a CNN–LSTM-based abnormal-behavior detector. He et al. [121] combined a BiLSTM–Attention-based cyberattack detector with simulation and semi-physical HIL evaluation, whereas Li et al. [102] used an FPGA-based HIL framework for real-time validation of automotive network-security strategies. These examples demonstrate that HIL/VIL can serve not only as a source of controlled attack scenarios and training data, but also as the closed-loop experimental environment in which AI-based detectors and their real-time cybersecurity performance are evaluated.

From this perspective, automotive cybersecurity validation can be represented as an attack-to-detection workflow. First, the relevant vehicle architecture and attack surface are identified. Second, the corresponding attack mechanism is reproduced through network manipulation, malicious communication, sensor perturbation, software modification, or other controlled injection mechanisms. Third, the HIL/VIL environment captures the resulting cyber–physical response through network traffic, ECU states, sensor measurements, actuator commands, and vehicle-dynamic variables. These synchronized data can then be used by AI-based intrusion or anomaly-detection models to distinguish nominal behavior from malicious or abnormal operation. Finally, detection performance should be evaluated together with real-time execution constraints and, where applicable, the physical safety impact of the attack. When mitigation or response mechanisms are available, the same HIL/VIL environment can also be used to evaluate whether containment, filtering, isolation, fallback, or recovery actions prevent attack propagation and unacceptable cyber–physical consequences.

Table 5 synthesizes this relationship by linking representative automotive architectures and attack surfaces to cyberthreats, HIL/VIL representations, observable data, AI-based detection approaches, and relevant evaluation criteria.

The mappings in Table 5 combine detection and validation approaches demonstrated in the surveyed literature with candidate approaches derived from the authors’ synthesis of the identified attack surfaces and observable variables. Accordingly, the representative citations associated with each row should not be interpreted as evidence that every listed detection or validation approach has been experimentally demonstrated within a single study.

The synthesis in Table 5 emphasizes that the appropriate detection strategy depends on both the attack surface and the observable variables available in the experimental platform. Network-only attacks may be detected from message timing, payload, or traffic statistics, whereas sensor, software, and cross-domain attacks may require correlation between network activity and physical vehicle behavior. HIL/VIL therefore provides a common experimental layer in which attack mechanisms, observable cyber–physical effects, and AI-based detection performance can be evaluated within the same controlled workflow. Finally, Section 6 summarizes the principal conclusions of the review and discusses remaining challenges and future research directions.

## 6. Conclusions and Future Perspectives

The increasing connectivity, software complexity, and architectural centralization of modern vehicles have substantially expanded the automotive cybersecurity attack surface. This review examined threats affecting not only traditional in-vehicle networks but also contemporary domain- and zonal-oriented architectures, Automotive Ethernet, V2X connectivity, cloud and backend services, OTA and software-update mechanisms, and sensor and perception systems. These developments demonstrate that automotive cybersecurity can no longer be evaluated solely at the level of individual ECUs or communication buses, because attacks may propagate across software, network, sensing, and physical-control domains.

HIL and VIL environments provide an important experimental foundation for evaluating these cyber–physical interactions under controlled and repeatable conditions. By combining real automotive hardware with simulated vehicle dynamics, communication interfaces, sensors, actuators, and operating environments, these platforms can reproduce representative attack scenarios while preserving realistic timing and closed-loop behavior. Digital-twin approaches can further complement HIL/VIL by providing synchronized virtual representations that support monitoring, scenario generation, and data-driven analysis. Together, these environments enable the effects of cyberattacks to be observed across network, ECU, sensor, actuator, and vehicle-dynamic layers without relying exclusively on costly or potentially unsafe full-vehicle experimentation.

AI-based intrusion and anomaly detection provides a complementary layer for interpreting the data generated through such experiments. The surveyed literature includes conventional machine-learning methods, temporal and attention-based deep-learning models, graph-based approaches, state-space models, generative methods, and hardware-oriented implementations targeting real-time deployment. However, evaluation should extend beyond classification accuracy to include false-alarm behavior, detection latency, robustness across vehicles and operating conditions, resource and power requirements, and resilience to adversarial manipulation. Detection should also be considered together with mitigation and response, particularly when the objective is to prevent an identified cyberattack from propagating toward safety-relevant vehicle functions.

An important finding of this review is that, although automotive cybersecurity, AI-based intrusion detection, and HIL/VIL validation are each active research areas, comparatively few studies integrate all three within a single experimental framework. The limited number of directly relevant studies identified in this review indicates that the integration of realistic cyberattack reproduction, synchronized cyber–physical data acquisition, AI-based detection, and closed-loop validation remains an emerging research direction. This gap is particularly evident for contemporary attack surfaces such as cloud and backend services, OTA software updates, heterogeneous CAN–Ethernet architectures, service-oriented communication, and physical-world perception attacks.

Future research should therefore move toward unified AI–HIL/VIL cybersecurity validation pipelines that connect attack generation, cyber–physical observation, detection, mitigation, and response within the same experimental workflow. Important directions include the development of open and standardized benchmarks containing synchronized network and physical signals, evaluation across multiple vehicle platforms and previously unseen attacks, integration of cloud, V2X, OTA, and perception-oriented attack scenarios, and systematic assessment of adversarial robustness. Greater attention is also required for real-time and resource-constrained deployment, explainability, continual adaptation to evolving threats, and alignment with automotive cybersecurity engineering and regulatory frameworks.

Overall, the convergence of automotive cybersecurity, AI-based detection, and HIL/VIL validation offers a promising foundation for the systematic evaluation of future connected and software-defined vehicles. The principal opportunity for the field is not simply to develop increasingly accurate detection algorithms, but to validate whether these mechanisms can detect, contain, and respond to realistic attacks before they produce unacceptable cyber–physical consequences.

## Figures and Tables

**Table 1:** Selected reported automotive cybersecurity incidents in recent years.

Year	Company/System	Reported Incident
2018	Volkswagen	Remote code execution was reported in the infotainment system of the Golf GTE.
2018	Honda	An improperly configured cloud server exposed the personal information of more than 50,000 users.
2019	Mercedes-Benz	An onboard application was compromised and reportedly enabled the theft of more than 100 vehicles.
2019	Toyota	Four security vulnerabilities were reported in the navigation system of the 2017 NX300 model.
2020	Mercedes-Benz	A total of 9765 vehicles were recalled because of software problems affecting the communication module.
2020	Volkswagen	An attacker reportedly obtained a vehicle key by exploiting the digital-signature transponder.
2021	QNX operating system	Security researchers disclosed multiple vulnerabilities affecting the automotive QNX operating system.
2022	Honda	A weakness in the rolling-code mechanism enabled replay of a previously transmitted keyless-entry command.
2023	Toyota	A cloud misconfiguration allowed unauthorized access to databases managed by Toyota Connected Corporation.
2023	Tesla	At Pwn2Own Vancouver, researchers demonstrated remote code execution in the in-vehicle infotainment system and transmitted CAN messages affecting other ECUs.
2024	Tesla	At Pwn2Own Automotive, the Synacktiv team successfully used a two-bug exploit chain against the Tesla infotainment system [23].
2024	Kia	A vulnerability in remote services allowed misuse of cloud APIs to perform vehicle operations such as locking, unlocking, and starting, potentially affecting more than one million vehicles.

**Table 2:** Summary of major literature review articles on automotive cybersecurity.

Year	Ref.	Attack Classification	Defense Classification	Main Contribution
2019	[69]	Availability, confidentiality, and data integrity	Not reported	Classified automotive attacks and the corresponding defense techniques.
2021	[70]	Autonomous control systems, driving components, and V2X communication	Security architecture, intrusion detection, and anomaly detection	Provided a systematic analysis of attack and defense strategies for autonomous vehicles.
2023	[64]	Sensor and perception attacks, safety violations, and attacker capabilities	Not reported	Reviewed vulnerabilities affecting sensors and perception systems in autonomous vehicles.
2023	[71]	In-vehicle network, remote, and firmware attacks	Lightweight IDS models and AI-based anomaly detection	Reviewed in-vehicle network security challenges from a protocol-oriented perspective.
2026	[11]	Vehicle-SOC threat models and data-driven cyberattack patterns	SOC analytics and AI-supported detection workflows	Proposed an automotive intelligence framework to support vehicle-SOC analysts.

**Table 3:** Representative automotive cybersecurity testbeds and their underlying platforms.

Year	Ref.	Test Platform	Testbed or Framework
2017	[85]	Software simulator	Testbed for Automotive Cybersecurity.
2018	[86]	Portable hardware/software testbed	Portable Automotive Security Testbed with Adaptability (PASTA).
2019	[76]	HIL testbed	Hardware-in-the-Loop-Based Automotive Embedded Systems Cybersecurity Evaluation Testbed.
2023	[87]	Software simulator	Cybersecurity Simulator for Connected and Autonomous Vehicles.
2024	[84]	Hybrid test platform	HackCar: A Test Platform for Attacks and Defenses on a Cost-Contained Automotive Architecture.
2024	[12]	HIL and fault-injection framework	Virtual Testing Framework for Real-Time Validation of Automotive Software Systems Based on HIL and Fault Injection.
2025	[75]	Vehicle-in-the-loop/HIL testbed	Cybersecurity Dynamometer Testbed for Vehicle-in-the-Loop Evaluation.

**Table 4:** Representative simulation, HIL, and VIL environments combined with AI-based methods for automotive cybersecurity evaluation.

Year	Ref.	Test Platform	Attack/Anomaly	AI-Based Method and Purpose	Study/Framework
2022	[88]	Automotive HIL test bench	Injected sensor and actuator faults	Hybrid CNN–LSTM for detection and classification of abnormal behavior from HIL-generated traces	Intelligent fault detection and classification for HIL-based automotive software testing.
2024	[66]	AV testbed and simulation	GNSS/GPS spoofing	Vehicle-behavior modeling and ML using temporal features for spoofing detection	GPS-IDS: anomaly-based GPS spoofing detection for autonomous vehicles.
2025	[102]	FPGA-based HIL framework	Automotive network attacks	Real-time HIL acceleration and evaluation of IDS/IPS security strategies	FAV-NSS: HIL framework for validating automotive network-security strategies.
2025	[121]	Simulation and semi-physical HIL platform	False-data-injection attacks on the lateral control system	BiLSTM–Attention-based detection using physically guided vehicle, steering-system, and actuator features	Cyber-attack detection for the lateral control system of a cloud-based intelligent connected vehicle.

**Table 5:** Synthesis of automotive attack surfaces, HIL/VIL representation, observable data, AI-based detection approaches, and evaluation criteria.

Architecture/ Attack Surface	Representative Threats	HIL/VIL Representation	Observable Signals/ Data	AI-Based Detection Approach	Evaluation Criteria/ Representative Sources
CAN/CAN FD/CAN XL	Injection, replay, masquerading, DoS, bus-off	Real or emulated CAN interfaces connected to ECUs or networked controllers; controlled message injection and traffic manipulation	CAN identifiers, payloads, timing, message frequency, ECU inputs/outputs	Sequence modeling, timing-based IDS, supervised or attention-based classification	Detection rate, false alarms, latency, attack classification; [90,98,101,102]
Automotive Ethernet/ gateways/ zonal networks	Spoofing, DoS, manipulated service traffic, cross-domain propagation, gateway compromise	Switched Ethernet links, gateway or zonal-controller models, heterogeneous CAN–Ethernet communication, service-oriented traffic	Packet/service traffic, communication timing, gateway events, ECU/network states	Traffic anomaly detection, supervised classification, temporal or service-behavior modeling	Cross-domain detection, gateway response, latency, propagation containment; [19,37,42]
V2X/C-V2X connectivity	Spoofing, replay, impersonation, message manipulation, DoS	Emulated or controlled V2X communication interfaces integrated with vehicle or VIL scenarios	V2X messages, timing, positioning data, vehicle state, communication events	Anomaly detection, temporal behavior modeling, position-consistency analysis	Detection accuracy, false alarms, robustness to mobility and operating conditions; [49,66,77]
Cloud/backend/ API/telematics	Unauthorized commands, authentication or authorization weaknesses, backend compromise, malicious cloud-to-vehicle requests	Emulated backend or telematics interfaces and controlled external commands connected to vehicle gateways or ECUs	API requests, telematics messages, ECU responses, network traffic, vehicle/control states	Behavioral anomaly detection and cross-layer correlation of off-board and in-vehicle events	Unauthorized-command detection, propagation to vehicle functions, response latency; [14,60]
OTA/firmware/ software supply chain	Malicious firmware, rollback, compromised update servers, unauthorized software modification	Controlled update process, firmware/version manipulation, emulated update infrastructure, ECU reflashing	Firmware version, update messages, authentication results, ECU state, network and control responses	Integrity/anomaly monitoring and behavioral detection of abnormal post-update operation	Update integrity, rollback prevention, successful containment and cyber–physical impact; [10,61,62]
Sensors/ perception/ cyber–physical interfaces	GNSS spoofing, sensor manipulation, falsified wheel-speed or control-relevant measurements	HIL/VIL sensor substitution or perturbation while maintaining closed-loop vehicle dynamics	Sensor measurements, actuator commands, controller states, vehicle-dynamic variables	CNN–LSTM, temporal anomaly detection, vehicle-behavior modeling	Detection accuracy, false alarms, detection latency, robustness across operating conditions; [66,88]

## Data Availability

No new data were created or analyzed in this study. Data sharing is not applicable to this article.

## Abbreviations

The following abbreviations are used in this manuscript:

ABS	Anti-lock Braking System
ADAS	Advanced Driver-Assistance Systems
AE	Automotive Ethernet
AI	Artificial Intelligence
CAN	Controller Area Network
CNN	Convolutional Neural Network
DL	Deep Learning
DoS	Denial of Service
DSRC	Dedicated Short-Range Communications
ECU	Electronic Control Unit
GNSS	Global Navigation Satellite System
GPS	Global Positioning System
GRU	Gated Recurrent Unit
HIL	Hardware-in-the-Loop
IDS	Intrusion Detection System
IVN	In-Vehicle Network
LIN	Local Interconnect Network
LSTM	Long Short-Term Memory
ML	Machine Learning
MOST	Media-Oriented Systems Transport
OBD-II	On-Board Diagnostics II
OTA	Over-the-Air
TCN	Temporal Convolutional Network
V2X	Vehicle-to-Everything
VIL	Vehicle-in-the-Loop
AUTOSAR	AUTomotive Open System ARchitecture
BSW	Basic Software
RTE	Runtime Environment
SOME/IP	Scalable service-Oriented MiddlewarE over IP
CSMS	Cyber Security Management System
SUMS	Software Update Management System
EMC	Electromagnetic Compatibility
